# Nutrient Intake Among Lactating Women With Overweight and Obesity in Norway: A Comparison With the Nordic Nutrition Recommendations 2023

**DOI:** 10.1111/jhn.70000

**Published:** 2025-01-06

**Authors:** Maria Fossli, Elisabeth A. Øhman, Malin Andal, Beate F. Løland, Kirsten B. Holven, Hilde K. Brekke

**Affiliations:** ^1^ Norwegian Research Centre for Women's Health Oslo University Hospital Oslo Norway; ^2^ Department of Nutrition, Institute of Basic Medical Sciences University of Oslo Oslo Norway; ^3^ Cluster for Research and Analysis of the Health Services Norwegian Institute of Public Health Oslo Norway; ^4^ Norwegian National Advisory Unit on Familial Hypercholesterolemia, Department of Endocrinology, Morbid Obesity and Preventive Medicine Oslo University Hospital Oslo Norway

**Keywords:** lactation, maternal, Nordic Nutrition Recommendations, nutrient intake, obesity, overweight, postpartum

## Abstract

**Background:**

During lactation, maternal requirements for many nutrients increase due to the physiological demands of breast milk production, reflected in dietary recommendations. BMI is negatively associated with dietary quality postpartum, and 40% of women in Norway have pre‐pregnancy overweight and obesity. Currently, there is limited data on dietary intake among lactating women in Norway and whether they meet nutritional requirements. We aimed to evaluate the nutrient intake in a study sample of lactating women with overweight and obesity, compared with the Nordic Nutrition Recommendations (NNR 2023).

**Methods:**

In this cross‐sectional analysis, we included baseline data from 112 lactating women with a pre‐pregnancy BMI of 25–35 kg/m^2^, participating in a weight loss and breastfeeding promotion intervention trial in Oslo, Norway. Data were collected at 2 weeks postpartum (subject characteristics, anthropometry and dietary supplement use), at 7 weeks postpartum (dietary assessment) and post‐weaning (retrospective dietary supplement use). Dietary data were obtained from a 4‐day dietary record before randomisation to dietary treatment for weight loss. Nutrient intake was compared to the dietary reference values for lactating women in NNR 2023. Increased risk of inadequate intake of micronutrients was assessed as the proportion of women with intakes below the average requirement (AR), with and without dietary supplements.

**Results:**

Mean ± SD BMI at 2 weeks postpartum was 30.7 ± 2.5 kg/m^2^. At 7 weeks postpartum the women reported a mean energy intake of 9.2 ± 2.0 MJ/day, with a higher intake of saturated fat and a lower intake of carbohydrate, dietary fibre and docosahexaenoic acid than recommended. The majority had an increased risk of inadequate intake of vitamin A (92%), folate (92%), vitamin D (84%), selenium (87%) and iodine (71%) from the diet alone. When dietary supplements were taken into account, ≥ 50% of the women still had an increased risk of inadequate intake of vitamin A, folate and selenium.

**Conclusions:**

The high proportion of lactating women with overweight and obesity failing to meet the newly updated Nordic Nutrition Recommendations highlights the need to raise awareness among new mothers and healthcare professionals about the increased maternal nutritional demands during lactation and hence, the importance of nutrient‐dense diets.

## Summary

- During lactation, maternal nutrient requirements increase to meet the demands of breast milk production and to ensure the health of both mother and infant.
- A large proportion of women with overweight and obesity in Norway failed to meet the Nordic Nutrition Recommendations 2023 for lactating women.
- The majority of lactating women with overweight and obesity in Norway had an intake below the average requirement (AR) for vitamins A and D, folate, selenium and iodine from their diet alone.

## 1 Introduction

The lactation period is a critical phase of the life cycle when women are particularly vulnerable from a nutritional perspective. During lactation, maternal requirements for many nutrients are increased in response to the physiological demands of breast milk production [1] and for ensuring the good health of both the mother and the infant [2]. The concentration of certain nutrients found in breast milk, that is, long‐chain omega‐3 fatty acids, vitamins A, E and K, iodine and selenium, is influenced by the mother's diet [3]. For some nutrients (e.g., folate, calcium, zinc and magnesium), the continuous supply for milk production may be at the expense of maternal reserves [2, 3, 4]. During the breastfeeding period, the dietary intake of the mother often receives little attention [5], as the nutritional and postnatal focus tends to be on the newborn, and the postpartum follow‐up of women's health is limited [6].

During lactation, the diet quality deteriorates for many women compared to pregnancy, with decreased fruit and vegetable intake and increased consumption of fat, sweets and snacks [7]. Inadequate dietary intake of several nutrients has been observed among lactating women in both high‐ and low‐income countries [8]. In addition, having a high BMI is a negative predictor of diet quality postpartum [9], and mothers with obesity report experiencing several barriers to healthy eating after delivery [10].

For lactating women, the 2023 update of the Nordic Nutrition Recommendations (NNR 2023) now includes average requirement (AR) values in addition to recommended intake (RI) values for micronutrients, whereas previous editions only provided RI values. The AR is a dietary reference value (DRV) estimated to meet the nutrient requirements of 50% of the individuals in a specific population. The RI, calculated as AR + 2 standard deviations (SD), is a DRV that includes safety margins estimated to meet the requirement for nearly the entire population.

The requirements for nutrients like protein, vitamins A, K and E, most B vitamins, calcium, selenium and iodine are elevated during lactation [1]. In Norway, lactating women are currently advised to follow the same dietary guidelines as the general population and to use a vitamin D supplement or cod liver oil [11]. Lactation increases energy requirements [1] and consuming a diet within these guidelines can enhance the likelihood of consuming increased amounts of essential nutrients.

In Norway, dietary data from national surveys are available for adults, children, infants and toddlers [12]. Additionally, dietary data in pregnant women are available from the large prospective population‐based Norwegian Mother and Child Cohort Study (MoBa) [13]. Although a few studies have examined the intake of individual micronutrients, such as iodine, among Norwegian lactating women [14, 15], a comprehensive investigation of their overall dietary intake is lacking.

The goal of this cross‐sectional analysis was to evaluate the dietary intake in a study sample of 112 lactating women with overweight and obesity in Norway. The primary aim was to compare the nutrient intake from the diet alone with the recently updated NNR 2023 for lactating women. The secondary aim was to examine the contribution of dietary supplements to the micronutrient and docosahexaenoic acid (DHA) intake.

## 2 Material and Methods

### 2.1 Study Design

**Figure 1:**
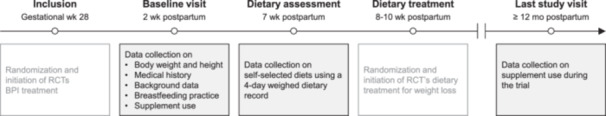
Timeline of data collection in this cross‐sectional analysis of nutrient intake from a weight loss and breastfeeding promotion intervention trial. BPI, breastfeeding promotion intervention.

This is a cross‐sectional analysis of baseline data collected in an RCT designed to investigate the effects of a dietary treatment for weight loss and a breastfeeding promotion intervention (BPI) on weight and cardiometabolic risk factors in a 2 × 2 factorial design at the Department of Nutrition, University of Oslo, Norway [16]. Data on subject characteristics were obtained at 2 weeks postpartum at the RCT's baseline visit, while a dietary assessment was conducted at 7 weeks postpartum, before the initiation of the RCT's dietary intervention (Figure 1).

At mean gestational week 28.1 ± 5.2, both inclusion in the RCT and allocation and initiation of the BPI treatment took place (Figure 1). BPI consisted of individualised guidance and support to follow the Norwegian breastfeeding recommendations [17]. Counselling was provided by experienced lactation consultants and is described in detail elsewhere [16]. The lactation consultants did not give any dietary advice. The RCT's baseline visit took place at 2 weeks postpartum at the study centre and included data collection on anthropometric measurements, background data, medical history and breastfeeding practice. A 4‐day weighed dietary record of self‐selected diets was conducted at 7 weeks postpartum. Randomisation and initiation of the RCT's dietary treatment for weight loss, which is described elsewhere [16], was performed after the participants returned their dietary records by email or mail.

### 2.2 Study Subjects

Pregnant women with a self‐reported pre‐pregnancy BMI of 25–35 kg/m^2^, residing in the Oslo area, were recruited between 30 January 2018 and 3 December 2021, through posters and flyers in community health centres and on social media. The inclusion criteria for the women were as follows: ability to read and write Norwegian, intention to breastfeed, no history of breast reduction surgery, singleton pregnancy and no chronic diseases or use of medications affecting lipid and glucose metabolism. The exclusion criteria included new chronic diseases or use of medication affecting lipid and glucose metabolism, pre‐eclampsia, drug‐treated gestational diabetes, abortion, stillbirth and short gestation (less than 36 weeks). In total, 156 women were included in the RCT, of which 119 completed the baseline visit. In this paper, we only included the women who reported breastfeeding exclusively or partially at 2 weeks postpartum, had no missing subject characteristic data and completed a 3‐ or 4‐day dietary record, resulting in a total of 112 women. Exclusive breastfeeding was defined as the infant receiving only breast milk, with the exception of vitamins, minerals and medicines, while partial breastfeeding was defined as the infant receiving breast milk in combination with any food or liquid [18]. Among the 112 women included in the current paper, 63 (56%) received the BPI treatment. The RCT named EVA (in Norwegian: Effekter av Vektnedgang og Amming [Effects of Weight loss and Lactation]) was approved by the Regional Ethical Committee of Oslo (2017/451), conducted in accordance with the Declaration of Helsinki and registered at ClinicalTrials.gov (NCT03580057). A written informed consent was obtained from all participants. The current paper was prepared in accordance with the Strengthening the Reporting of Observational Studies in Epidemiology (STROBE) checklist for cross‐sectional studies (www.strobe-statement.org).

### 2.3 Measurements

#### 2.3.1 Dietary Intake

The women were informed by a clinical dietitian to weigh all foods and beverages consumed for four consecutive days including at least 1 weekend day, while continuing their habitual diets. The participants received written instructions on how to record the intake of each food item, including the amount consumed, time of consumption, methods of preparation, brand name and recipes of composite dishes. These instructions were followed by a detailed example of a dietary record. Kitchen scales were provided for the women who did not have scales at home. The use of household measurements for reporting portion sizes was accepted to minimise the burden of weighing and in situations where kitchen scales were not available. Nutrient intake was calculated in Dietist Net Pro (Kostdata.se, Bromma, Sweden) with the Norwegian Food Composition Table 2020 (http://www.matvaretabellen.no).

#### 2.3.2 Dietary Supplements

We collected data on dietary supplement use for 102 out of the 112 women (91%) using interviews. These data were obtained at 2 weeks postpartum for 43 women (42%) who reported their current use. For the remaining women (*n* = 59, 58%), we utilised data from the last study visit of the RCT, post‐weaning (at ≥ 12 months postpartum), where they retrospectively reported their supplement use during the lactation period (Figure 1). The women were instructed to specify the types of dietary supplement used (e.g., multivitamins and minerals, cod liver oil, omega‐3 fatty acids, vitamin D, iron etc.) and whether the supplements were aimed at lactating women or not. Brand names were not recorded; however, if different brands had varying nutrient content, the brand with the lowest nutrient content was used in the calculations.

#### 2.3.3 Anthropometric Measures

At 2 weeks postpartum, anthropometric measurements were performed at the study centre by a clinical dietitian after an overnight (≥ 10‐h) fast. Height was measured barefoot to the nearest 0.1 cm using a wall‐mounted stadiometer (Seca 264, Hamburg, Germany). Weight was measured barefoot to the nearest 0.1 kg with lightweight clothing on Seca mBCA 515 (Hamburg, Germany). BMI (kg/m^2^) was categorised as overweight (25.0–29.9), obesity class I (30–34.9), obesity class II (35–39.9) or obesity class III (≥ 40).

### 2.4 Data Analysis and Statistics

Data are presented as means ± SD or as medians with the first and third quartiles (Q1, Q3). The statistical analyses were carried out in IBM SPSS Statistics for Windows version 26.0 (Armonk, NY).

#### 2.4.1 Nutrient Intake

The nutrient intake was compared to NNR 2023 for lactating women [1]. In the NNR, recommendations for macronutrients are given as ranges or specific percentages of total energy consumed (E%). The recommendation for dietary fibre is given in g and for DHA in mg. The intake of micronutrients was primarily compared to AR, as AR is used to assess the adequacy of micronutrient intake in specific groups [1]. The percentage of individuals with an intake below the AR indicates the proportion with an increased risk of inadequate intake. We additionally calculated the proportion of women with a micronutrient intake between AR and RI and those meeting or exceeding RI. Provisional AR was utilised when AR had not been established, while adequate intake (AI) was applied when RI had not been determined [1].

#### 2.4.2 Evaluation of Underreporting

Reported energy intake (EI) compared to the estimated energy requirement (EER) is a common method to assess the degree of underreporting in dietary assessments [19]. We investigated the extent of misreporting of EI by calculating the relative (%) difference between EER at 2 weeks postpartum and reported EI at 7 weeks postpartum. EER was determined by multiplying the basal metabolic rate (BMR) by a physical activity level (PAL). BMR was predicted by Henry's equation for women aged 30–60 years based on body weight and height, and found to be specifically suitable for this group of women [20]. We calculated EER for three different activity levels using PAL values 1.4, 1.6 and 1.8, representing sedentary, moderate or average, and active lifestyles, respectively [1]. EER was corrected for the energy cost of lactation minus the average energy mobilisation from fat stores (0.72 MJ/day) [21]. The energy cost of milk production was calculated for exclusive breastfeeding (749 g/day) and partial breastfeeding (492 g/day), with a milk energy density of 2.8 kJ/g and an energetic efficiency of 0.8 [21, 22].

## 3 Results

### 3.1 Study Participants

In total, we included 112 lactating women who completed a dietary record, with 108 (96%) recording 4 days, and 4 (4%) recording 3 days. At 2 weeks (mean 15.4 ± 5.0 days) postpartum, the mean age was 33.6 ± 3.9 (range: 25.2–45.3) years and the mean BMI was 30.7 ± 2.5 (range: 26.1–37.7) kg/m^2^ (Table 1). The majority of our study population had 5 or more years of education beyond high school (61%) and practiced exclusive breastfeeding (86%). At 2 weeks postpartum, the estimated EER for our participants was 12.1 ± 0.7 MJ/day, assuming a moderate physical activity level (PAL = 1.6).

**Table 1:** Subject characteristics at 2 weeks postpartum in a study sample of 112 lactating women with overweight and obesity.

Age (years)	33.6 ± 3.9
Pre‐pregnancy body weight^1^ (kg)	82.1 ± 9.3
Body weight (kg)	87.2 ± 9.3
Height (cm)	168.4 ± 6.4
BMI (kg/m^2^)	30.7 ± 2.5
BMI category, *n* (%)	
Overweight	47 (42)
Obesity class I	56 (50)
Obesity class II	9 (8)
Primiparous, *n* (%)	55 (49)
Breastfeeding, *n* (%)	
Exclusive	97 (86)
Partial	16 (14)
Diet‐treated gestational diabetes, *n* (%)	7 (6)
Education, *n* (%)	
High school or lower	4 (3)
< 5 years beyond high school	40 (36)
≥ 5 years beyond high school	68 (61)
Married or cohabitating, *n* (%)	105 (94)
BMR^2^ (MJ/day)	6.5 ± 0.4
EER^3^ (MJ/day)	
Sedentary PAL 1.4	10.8 ± 0.6
Moderate PAL 1.6	12.1 ± 0.7
Active PAL 1.8	13.4 ± 0.8

*Note:* Normally distributed values are presented as mean ± SD, while categorical variables are presented as *n* (%).

Abbreviations: BMR, basal metabolic rate; EER, estimated energy requirement; PAL, physical activity level.

^1^ Self‐reported.

^2^ BMR predicted with Henry's equation for women aged 30–60 years based on body weight and height [20].

^3^ EER was corrected for the energy cost of lactation minus the average energy mobilisation from fat stores [21, 22].

### 3.2 Nutrient Intake

At 7 weeks postpartum, the mean EI was 9.2 ± 2.0 MJ/day. At the group level, the women had a higher intake of saturated fat (15 energy percentage [E%]), and a lower intake of carbohydrate (42 E%), dietary fibre (2.6 g/MJ) and DHA (160 mg/day) than recommended (Table 2). Sixty‐four women (57%) met the specific recommended protein intake of 0.83 g/kg bodyweight with an addition of 13 g/day during the first 6 months of lactation. Furthermore, 78 women (70%) had a lower energy intake from carbohydrates, while 99 women (88%) had a higher energy intake from saturated fat than recommended. Less than half of the subjects met the recommendations for omega‐3 fatty acids, DHA and dietary fibre (Table 2).

**Table 2:** Dietary macronutrient intake and the proportion (%) meeting the NNR 2023 recommendations in a study sample of 112 lactating women with overweight and obesity at 7 weeks postpartum.

			NNR^1^	Meeting NNR^1^
	Mean ± SD	Median (Q1, Q3)		*n* (%)
Protein (E%)	16.8 ± 2.5	16.7 (15.1, 18.5)	10–20	99 (88)
Fat (E%)	38.3 ± 5.8	37.8 (34.7, 41.7)	25–40	71 (63)
Saturated fat (E%)	14.5 ± 3.6	14.5 (12.4, 16.6)	< 10	13 (12)
Monosaturated fat (E%)	14.7 ± 3.1	14.1 (12.7, 16.7)	10–20	92 (82)
Polyunsaturated fat (E%)	6.0 ± 1.5	5.9 (5.0, 6.7)	5–10	83 (74)
Essential fatty acids (E%)^2^	5.5 ± 1.4	5.4 (4.4, 6.2)	≥ 5	75 (67)
Omega‐3 fatty acids (E%)^3^	1.1 ± 0.5	1.0 (0.8, 1.3)	≥ 1	54 (48)
DHA (mg)	261 ± 265	160 (60, 453)	200	49 (44)
Carbohydrate (E%)	41.8 ± 5.6	41.8 (38.2, 45.7)	45–60	34 (30)
Added sugar (E%)	7.9 ± 3.7	7.3 (5.0, 10.7)	< 10^4^	78 (70)
Alcohol (E%)	0.9 ± 2.0	0.0 (0.0, 0.8)	—	—
Dietary fibre (g/MJ)	2.6 ± 0.6	2.5 (2.1, 3.0)	≥ 3	25 (22)
Dietary fibre (g)	23.9 ± 7.9	23.0 (18.2, 27.7)	≥ 25	44 (39)

*Note:* Nutrient intake was calculated based on a 4‐day dietary record without dietary supplements.

Abbreviations: AA, arachidonic acid; ALA, alpha‐linoleic acid; DHA, docosahexaenoic acid; DPA, docosapentaenoic acid; E%, percentage of total energy intake; EPA, eicosapentaenoic acid; LA, linoleic acid; NNR, Nordic Nutrition Recommendations.

^1^ Recommended intake range and recommended intake of macronutrients for lactating women 0–6 months postpartum in NNR 2023 [1].

^2^ DHA, DPA, EPA, ALA, LA and AA.

^3^ DHA, DPA, EPA and ALA.

^4^ In NNR 2023: Added/free sugar.

The proportion of women with an intake below AR, and thus with an increased risk of inadequate intake, was particularly high for vitamin A (92%), folate (92%), vitamin D (84%), selenium (87%) iodine (71%), vitamin C (48%) and riboflavin (48%) (Table 3).

**Table 3:** Dietary micronutrient intake and the proportion (%) with an intake below AR, between AR and RI, and meeting RI in a study sample of 112 lactating women with overweight and obesity at 7 weeks postpartum.

					< AR	AR – RI	≥ RI
	Mean ± SD	Median (Q1, Q3)	AR^1^	RI^1^	*n* (%)	*n* (%)	*n* (%)
Vitamin A, RE	656.9 ± 314.3	602.8 (427.4, 793.5)	1060	1400	103 (92)	9 (8)	0 (0)
Vitamin D (µg)	4.8 ± 3.0	4.4 (2.4, 6.5)	7.5	10	94 (84)	11 (10)	7 (6)
Vitamin E, α‐TE	14.1 ± 5.0	13.5 (10.2, 17.1)	10^2^	12^3^	23 (21)	17 (15)	72 (64)
Thiamine (mg/MJ)	0.18 ± 0.06	0.17 (0.13, 0.20)	0.07	0.1	1 (1)	1 (1)	110 (98)
Riboflavin (mg)	1.7 ± 0.7	1.6 (1.3, 2.1)	1.6	2.0	54 (48)	28 (25)	30 (27)
Vitamin B6 (mg)	1.6 ± 0.5	1.4 (1.2, 1.8)	1.4	1.7	46 (41)	28 (25)	38 (34)
Vitamin B12 (µg)	6.2 ± 2.7	5.9 (4.1, 7.6)	4.2^2^	5.5^3^	29 (26)	21 (19)	62 (55)
Folate (µg)	258.9 ± 90.4	238.9 (201.2, 304.3)	380	490	103 (92)	6 (5)	3 (3)
Vitamin C (mg)	89.1 ± 48.3	76.5 (52.2, 119.9)	75	155	55 (49)	47 (42)	10 (9)
Calcium (mg)	1011.7 ± 388.0	969.2 (740.8, 1178.2)	800	950	34 (30)	18 (16)	60 (54)
Iodine (µg)	139.0 ± 73.8	117.6 (89.9, 163.2)	160^2^	200^3^	79 (71)	17 (15)	16 (14)
Iron (mg)	10.2 ± 3.2	9.5 (8.0, 12.1)	9	15	42 (38)	63 (56)	7 (6)
Selenium (µg)	49.3 ± 18.2	46.7 (36.7, 60.6)	70^2^	85^3^	97 (87)	11 (10)	4 (3)
Magnesium (mg)	340.7 ± 94.7	327.67 (263.1, 400.9)	240^2^	300^3^	17 (15)	21 (19)	74 (66)

*Note:* Nutrient intake was calculated based on a 4‐day dietary intake without dietary supplements.

Abbreviations: AI, adequate intake; AR, average requirement; RE, retinol equivalents; RI, recommended intake; TE, tocopherol equivalents.

^1^ Nordic Nutrition Recommendation 2023 for lactating women [1].

^2^ Provisional AR is calculated as 0.8 times the provisional recommended intake and is used when an AR cannot be determined. Provisional AR has larger uncertainty than AR.

^3^ AI is based on observed intakes in healthy people or approximations from experimental studies and is used when RI cannot be determined.

### 3.3 Dietary Supplements

**Figure 2:**
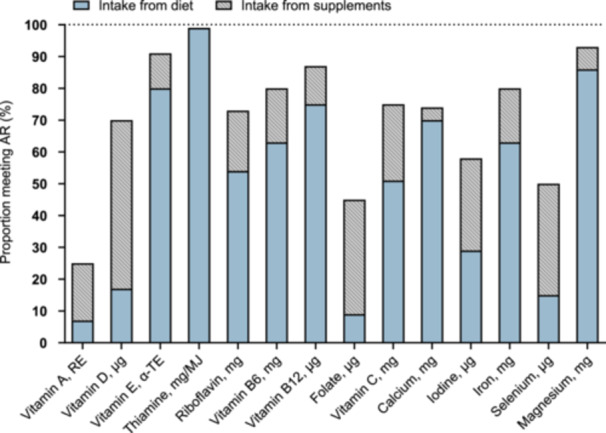
The proportion (%) of women (*n* = 102) with a micronutrient intake meeting or exceeding the average requirement (AR) for lactating women with and without dietary supplements.

Out of 102 women with data on the use of dietary supplements, 67 (66%) used at least one dietary supplement during lactation. Multivitamins and minerals were the most commonly used supplements, followed by fish oil/omega‐3 fatty acids, used by 40% and 37%, respectively (Table 4). The proportion of the 102 women with a micronutrient intake meeting or exceeding AR with and without dietary supplements is shown in Figure 2. When the nutrient intake from diet and dietary supplements was compiled, a high proportion of the women still had an intake below the AR for vitamin A (75%), folate (55%), selenium (50%) and iodine (42%). For DHA, 44% out of the 102 women met or exceeded the recommendation of 200 mg/day during lactation from their diet alone, while the proportion was 73% when the intake from dietary supplements was considered.

**Table 4:** Dietary supplements used during lactation in a study sample of 102 lactating women with overweight and obesity.

	*n* (%)
Multivitamins and minerals	41 (40)
Fish oil/omega‐3 fatty acids	38 (37)
Iron	16 (16)
Vitamin D	12 (12)
Cod liver oil	8 (8)
Iodine	5 (5)
Magnesium	4 (4)
Multivitamins	1 (1)
B12	1 (1)
Others	3 (3)

*Note:* Data on dietary supplement use were obtained at 2 weeks postpartum for 43 women (42%) and retrospectively, ≥ 12 months postpartum, for 59 women (58%) using interviews.

### 3.4 Evaluation of Underreporting

Based on a PAL value of 1.6, the mean ± SD underreporting of EI at 7 weeks postpartum was 24% ± 17%, with further details provided in Table S1.

## 4 Discussion

To our knowledge, this is the first analysis of the comprehensive nutrient intake among lactating women with overweight and obesity in Norway. We compared the nutrient intake of 112 mothers with the NNR 2023 for lactating women. The women had a higher intake of saturated fat and a lower intake of carbohydrate, dietary fibre and DHA than recommended. The majority of the women had an increased risk of inadequate intake of vitamins A and D, folate, selenium and iodine from the diet alone. Even when dietary supplements were considered, 50% or more of the women had an increased risk of inadequate intake of vitamin A, folate and selenium.

Our findings align with existing data on the general female Norwegian population, which also show a low intake of carbohydrate, dietary fibre, vitamin D and folate [23]. In addition, our findings share many similarities with Swedish studies in women with overweight and obesity, where the majority were breastfeeding, at 8–12 weeks postpartum [24, 25]. These women also had a higher intake of saturated fat and lower intake of dietary fibre, vitamin D and folate than recommended.

A low intake of foods rich in dietary fibre is common among lactating and postpartum women [5, 26, 27]. A high dietary fibre intake is associated with a decreased risk of non‐communicable diseases, such as coronary heart disease, type 2 diabetes and colorectal cancer, as well as maintaining a healthy body weight [28, 29]. Our findings suggest that lactating women with overweight and obesity could benefit from increasing their consumption of fibre‐rich foods.

Although the women in the present study had a relatively high median DHA intake compared to lactating women in other countries [30], they did still not meet the recommendation. In early life, DHA is important for the development of the central nervous system, including the brain [31]. Exclusively breastfed infants rely on the DHA content in their mother's milk and lactating women are recommended a daily intake of 200 mg [1, 3, 32]. An Icelandic study found that mothers consuming cod liver oil had significantly higher breast milk levels of DHA [33]. Both fish oil/omega‐3 fatty acids supplements and cod liver oil were important sources of DHA among the women in the present study and were altogether used by 47% of our women. Meeting the recommendation of DHA is not only beneficial for the breastfed infant, but may also reduce the risk of CVD in the mother [34].

Insufficient dietary intake of micronutrients has been observed among lactating women in both European and non‐European populations [8, 24, 25, 35, 36]. Durham et al. [37] reported that at 6–9 weeks postpartum, breastfeeding women with overweight in the United States were at risk for inadequate intakes of vitamins A, E, C and folate when compared with the US dietary reference intakes. More recently, the intake of folate, vitamins A, D and C among 180 lactating women with normal weight in a European multicenter cohort did not meet the European DRVs for lactating women [38].

For vitamin A, the doubling of AR compared to non‐lactating women makes it especially challenging to reach this level through the diet alone [39]. Currently, the content of vitamin A in general multivitamin supplements, as well as those aimed at lactating women, does not meet the AR, highlighting the importance of dietary intake. To date, the high requirement for vitamin A is undercommunicated in dietary advice from Norwegian health authorities [11, 40].

While folate supplementation is recommended before and during the first months of pregnancy [1, 41], postpartum folate intake often decreases due to reduced supplement use [42]. During lactation, folate requirements remain higher than in non‐pregnant women, but this is also underemphasised by the Norwegian health authorities [11, 40]. It is concerning that only 8% of the women in the current study met the AR for folate through the diet alone and 45% when dietary supplements were considered. A sufficient intake of folate protects against megaloblastic anaemia and deficiency is associated with an increased risk of CVD in the mother [41].

Similarly, low selenium intake has been associated with an increased risk of CVD in addition to cancer and all‐cause mortality [43]. The lactating women in our study had a low intake of selenium, consistent with the general Nordic female population [43]. Maternal selenium intake correlates with breastmilk content, and low intake is associated with a higher risk of infant infections and adverse neurodevelopment [43, 44, 45]. In a Norwegian study, 97%–99% of postpartum women had plasma selenium levels below what was previously considered ‘replete status’ of 124 µg/L [44]. While dietary intake data was not available from the latter study, 87% of the women in the present study had an increased risk of inadequate intake of selenium from the diet alone and 50% when dietary supplements were considered.

A low intake of iodine may affect the maternal thyroid gland function, and importantly, breastfed infants are dependent on a sufficient maternal intake of iodine for optimal growth and neurological development [46]. In two Norwegian studies focusing on iodine, lactating women had inadequate intakes of iodine from diet and supplements combined [14, 15], in line with our findings. One of the studies also investigated and found suboptimal iodine concentrations in breastmilk [14].

Meeting the average vitamin D requirement from diet alone is challenging [8, 30]. Although Norwegian health authorities advise lactating women to use a dietary supplement with vitamin D [11], only 61% in the present study reported doing so. Among the 64 supplement users, nearly all (93%) used a supplement containing vitamin D. Surprisingly, only 8% used cod liver oil, which is traditionally recommended as a source of vitamin D in Norway [11]. The use of vitamin D supplementation contributed greatly to the proportion of women reaching the AR (70%) when combined with the dietary intake, compared to only 16% meeting the AR through the diet alone.

The findings in this study underscore the need for providing guidance to lactating women on how to increase the intake of dietary fibre, DHA, iodine, vitamin A, folate, selenium and vitamin D from food. Our results also highlight the need for healthcare professionals to routinely recommend vitamin D supplementation to lactating women in Norway in line with the national advice. Although food is the preferred source of nutrients, supplements such as multivitamin and minerals and DHA may be considered if the intake of nutrients from the diet alone is inadequate. The gaps between actual intakes and the increased maternal nutritional requirements during lactation require more attention and targeted interventions.

At 7 weeks postpartum, many women are still adjusting to the major life changes associated with motherhood. Factors such as sleep deprivation, stress and limited resources to care for themselves and their newborn may influence dietary intake [47]. Women with obesity face additional barriers to healthy eating postpartum [10], potentially increasing their risk of future weight gain and more pronounced overweight and obesity with each pregnancy. Providing targeted support during the postpartum period is important for improving maternal health outcomes.

Underreporting of EI is common in dietary assessments and is associated with factors, such as high BMI, female sex, low educational level and high socioeconomic status [19, 48, 49]. In our study, we estimated that ∼24% of the reported EI was underreported, consistent with findings in Swedish lactating women with overweight and obesity, where total energy expenditure was measured using the doubly labelled water method [50]. Underreporting of EI in dietary records can result from underrecording, undereating or both [19, 51]. Because underreporting of EI typically leads to underreporting of nutrient intakes, the true nutrient intake among our participants may be higher than calculated. This assumes equal underreporting of all foods and beverages. However, selective underreporting is common and rather leads to underestimated intakes of nutrients found in foods perceived as unhealthy or high in energy [19]. Nevertheless, adjusting for an estimated 24% underreporting of EI still leaves over 50% of women with dietary intakes (excluding supplements) below the AR for vitamin A, folate, selenium, vitamin D and iodine. Thus, it is likely that our findings are not a result of underreporting but rather an intake of nutrients well below what is desirable.

A strength of this study is the comparison of micronutrient intake with newly published life‐stage AR values specific for lactating women in the NNR 2023. To our knowledge, this is the first study to investigate the overall nutrient intake, including both macro‐ and micronutrients, among lactating women in Norway. Our results are relevant to many new mothers, as 40% of women in Norway have pre‐pregnancy overweight or obesity [52]. The majority of our participants were exclusively breastfeeding and highly educated residing in in the Oslo area, factors associated with healthy eating habits [9, 23, 53]. Thus, the nutrient intake may be even more unfavourable in a more diverse population. A limitation of using a 4‐day dietary record is that it may not capture foods consumed irregularly, such as fish. This could potentially lead to an underestimation of the habitual intake of important nutrients like DHA, vitamins A and D, iodine and selenium. Another consideration is the comparison of the micronutrient intake to AR. When comparing the intake to RI, even fewer women meet this level, as RI is estimated to cover the requirement for nearly the whole population.

Optimally data on maternal body weight, breastfeeding status and supplement use should have been obtained at the same time point as the dietary records. EER was calculated using body weight measured 5 weeks before dietary assessment. However, given an expected weight loss of a maximum of 3 kg [16], the estimated underreporting of EI would remain very similar (23% vs. 24%). Data on breastfeeding status for all 112 women at various time points later in the RCT [16] confirm that breastfeeding status at 7 weeks postpartum was unchanged. In some cases, the dietary supplement use at 2 weeks postpartum and the use reported retrospectively post‐weaning may deviate from the actual use at 7 weeks postpartum. This limitation should be considered when interpreting nutrient intake including dietary supplements.

Future studies should include food group data to better understand how to develop specific food‐based dietary guidelines to improve nutrient intake in this group of women. It is also important to examine the nutrient intake among lactating women with low and normal BMI and in a more diverse study population.

## 5 Conclusion

A high proportion of Norwegian lactating women with pre‐pregnancy overweight and obesity failed to meet the current Nordic Nutrition Recommendations at 7 weeks postpartum. Our findings underscore the need to raise awareness among new mothers and healthcare professionals about the increased maternal nutritional demands during lactation. Eating a nutrient‐dense diet in line with NNR 2023 can also be beneficial in minimising retention of pregnancy weight and preventing weight gain postpartum, particularly among women with overweight and obesity. Incorporating maternal dietary guidance as part of breastfeeding advice in postnatal care could be a proactive step to increase the intake of important nutrients with the potential of benefiting both mothers and their breastfed infants at a critical time in the life cycle.

## Author Contributions

H.K.B. and E.A.Ø. designed the research. M.F., E.A.Ø. and H.K.B. conducted the research. M.F. and M.A. analysed data. M.F. drafted the paper and had primary responsibility for its final content. E.A.Ø., B.F.L., K.B.H. and H.K.B. contributed to the review and editing of the manuscripts. All authors read and proved the final manuscript.

## Ethics Statement

The main study was approved by the Regional Ethical Committee of Oslo (2017/451) and registered at ClinicalTrials.gov (NCT03580057).

## Conflicts of Interest

Kirsten B. Holven has received personal fees from Sanofi, none of which is related to the content of this manuscript. The other authors declare no conflicts of interest.

## Transparent Peer Review

The peer review history for this article is available at https://www.webofscience.com/api/gateway/wos/peer-review/10.1111/jhn.70000.

## Supporting information

Supporting information.

## Data Availability

The data that support the findings of this study are available on request from the corresponding author. The data are not publicly available due to privacy or ethical restrictions.
