# Supplementary material for: Nutrient Intake Among Lactating Women With Overweight and Obesity in Norway: A Comparison With the Nordic Nutrition Recommendations 2023
Source: J Hum Nutr Diet. 2025 Jan 6;38(1):e70000. doi: 10.1111/jhn.70000 (PMC11704453; doi:10.1111/jhn.70000)
Supplement: Supplementary file 1 — Supporting information. [file JHN-38-0-s001.docx]

| **SUPPLEMENTARY TABLE 1**  Estimated energy requirement^1^ and relative misreporting^2^ (%) of energy intake based on different physical activity levels in a study sample of 112 lactating women with overweight and obesity. | | | | | | | | |
| --- | --- | --- | --- | --- | --- | --- | --- | --- |
|  |  | EER (MJ/d) | | |  | (EER-EI)/EER | | |
| PAL |  | Mean | ± SD | Range |  | Mean | ± SD | Range |
| 1.4 |  | 10.8 | ± 0.6 | (9.3 ‒ 12.2) |  | 15% | ± 19% | (-44% ‒ 53%) |
| 1.6 |  | 12.1 | ± 0.7 | (10.5 ‒ 13.7) |  | 24% | ± 17% | (-30% ‒ 60%) |
| 1.8 |  | 13.4 | ± 0.8 | (11.7 ‒ 15.2) |  | 32% | ± 15% | (-17% ‒ 62%) |

Abbreviations: EER, estimated energy requirement; EI, energy intake; PAL, physical activity level.
^1^ EER was calculated based on weight and height measured at 2 weeks postpartum with BMR predicted with Henry's equation for women aged 30-60 (1). EER was corrected for the energy cost of lactation minus the average energy mobilization from fat stores. EI was calculated based on a 4-day dietary record at 7 weeks postpartum (2, 3).
^2^ A negative value indicates over-reporting while a positive value indicates underreporting.

1. Halland Nesse S, Ottestad I, Winkvist A, Bertz F, Ellegård L, Brekke HK. Predictive equations for estimating resting energy expenditure in women with overweight and obesity at three postpartum stages. J Nutr Sci. 2020;9:e31.

2. Cloetens L, Ellegård L. Energy - a scoping review for the Nordic Nutrition Recommendations 2023 project. Food Nutr Res. 2023;67.

3. Butte NF, King JC. Energy requirements during pregnancy and lactation. Public Health Nutr. 2005;8(7a):1010-27.
